# Effect of Anatomical Customization of the Fiber Post on the Bond Strength of a Self-Adhesive Resin Cement

**DOI:** 10.1155/2017/5010712

**Published:** 2017-07-13

**Authors:** Adricyla Teixeira Rocha, Leticia Machado Gonçalves, Ana Júlia de Carvalho Vasconcelos, Etevaldo Matos Maia Filho, Ceci Nunes Carvalho, Rudys Rodolfo De Jesus Tavarez

**Affiliations:** Post-Graduation Department, CEUMA University, São Luís, MA, Brazil

## Abstract

**Aim:**

The aim of the study was to evaluate, by means of the push-out test, the effect of the anatomical customization of the fiber post on the bond strength of a self-adhesive resin cement.

**Methods:**

Twelve endodontically treated, human, upper central incisors were randomly divided into two groups (*n* = 6): control (glass fiber posts cemented with Relyx® U200) and customized (glass fiber posts anatomically customized with translucent composite resin cemented with Relyx U200). The roots were sectioned into three slices, cervical, middle, and apical, and photographed with a digital camera attached to a stereomicroscopic loupe. The images were analyzed by software, for evaluation of the cement line. The slices were subsequently submitted to the push-out test until the post had completely extruded, and the fracture mode was analyzed with a stereomicroscopic loupe.

**Results:**

The results showed significant differences between the groups in the different root thirds in relation to the area occupied by air bubbles (*p* < 0.05). Bond strength, when all the thirds are considered, was 8.77 ± 4.89 MPa for the control group and 16.96 ± 4.85 MPa for the customized group.

**Conclusion:**

The customized group showed greater bond resistance than the control group and a more uniform cement layer.

## 1. Introduction

Despite present-day advances in Dentistry, with the incorporation and development of new restoration materials and techniques, big challenges still exist in terms of the rehabilitation of endodontically treated teeth, particularly in cases where the root canal is spacious and/or fragile [1, 2]. For many years, cast metal cores were regarded as the main option for the rehabilitation of endodontically treated teeth with weakened crown structure [3, 4]. However, in addition to not being esthetically satisfactory, this type of intraradicular retainer requires greater clinic time to fabricate it and wear and tear on the already fragile crown structure [5]. Another important factor is that, due to the metal's high elastic modulus when compared to that of root dentin, the core transfers a large part of the masticatory forces received directly to the root, which may result in fractures [6, 7].

Accordingly, prefabricated glass fiber posts were developed which, having an elastic modulus similar to root dentin and to resin cement [8], make it possible for a mechanically uniform unit to form that distributes masticatory load and protects the tooth remnant [9, 10]. Among other advantages, the final esthetic obtained should be mentioned, as well as less wear and tear on the tooth remnant and adhesion to the root dentin when used together with adhesive systems and resin cements [10, 11].

Being prefabricated, the glass fiber posts do not always adapt to the format and diameter of the root canals, a particularly important aspect for teeth with spacious or fragile root canals [12], If proper adaptation does not ensue, the resin cement line will be thick, [13] which may increase the polymerization pressure at the dentin/cement and post/cement interfaces and help to form bubbles and adhesion flaws [12, 13].

In an attempt to improve glass fiber post adaptation in cases of spacious root canals, one of the techniques proposed is the fabrication of anatomically customized posts [14]. This technique consists of customizing the prefabricated glass fiber post via the molding of the root canal with the direct application of composite resin [14, 15]. By increasing the adaptation of the post to the walls of the root canal, this technique should make it possible to form a thin layer of resin cement and, consequently, provide favorable conditions for retaining the post while the risk of adhesion failure would be reduced [12, 16–18].

It can be seen that, despite the clinical advantages of the customization technique, the literature is still rather lacking in terms of the increase in bond strength at the cement/post adhesive interface. Thus, the aim of the study was to evaluate the effect of the customization of the glass fiber post on the bond strength of a self-adhesive resin cement and to analyze the cement line of the adhesive interface. The null hypothesis is that there is no difference in the bond strength of anatomically customized or conventional posts at the cement/post adhesive interface and that the uniformity of the cement line has no impact on the bond strength of the self-adhesive resin cement.

## 2. Materials and Methods

### 2.1. Selection and Preparation of Teeth

The research project was approved by the ethics committee at Ceuma University (Protocol number 833.094).

Twelve human central incisors were collected, having straight roots, fully formed apexes, free from any type of cervical damage (caries, erosion, or abrasion) or previous endodontic treatment. The teeth were radiographed and those having tortuous and/or calcified canals were excluded. They were cleaned and stored in a solution of 0.1% thymol at 4°C.

The crowns were sectioned below the amelocemental junction using a double-faced diamond disk (KG Sorensen; Cotia, SP, Brazil) connected to a straight handpiece operating at low rotation and constantly cooled. The length of the roots was standardized at 18 mm (±1 mm). The cervical diameters of the root canals were measured in the medial-distal and buccolingual directions, with the aid of a digital caliper (Mitutoyo MTI Corporation, Tokyo, Japan), and root canals with a cervical diameter of 1.4 mm (±0.1 mm) were selected.

The working length (AWL) for each tooth was determined by introducing a K-type n°. 10 endodontic file (Maillefer, Dentsply Ind. e Com. Ltda., Petrópolis, RJ, Brazil) into the root canal until the tip of the file could be seen at the apical foramen and then subtracting 1 mm from the measurement obtained.

For the instrumentation of the canal, 10 mL of 1% sodium hypochlorite was used with instrumentation using a Reciproc R50 (VDW, Munich, Germany), and a Silver Reciproc Motor (VDW).

In an attempt to simulate wide root canals, these were prepared in a “preformed” sequence of #1, #2, and #3 burs for the post system used (White Post DC, FGM. Joinville, Santa Catarina, Brazil) in low rotation, inserted to a length of 13 mm. After these procedures, irrigation was carried out with 1 mL of EDTA (Fórmula e Ação, São Paulo, SP, Brazil) for three minutes. The canals were vacuum-dried using cannulas (Ultradent Products Inc., Salt Lake City, Utah, USA), the drying being complemented using absorbent paper cones (Dentsply Maillefer, Ballaigues, Switzerland).

### 2.2. Experimental Design

The 12 prepared roots were randomly divided, according to the type of post to be used, into two groups (*n* = 6): control group (noncustomized posts) and the customized group. Both groups used #1 glass fiber posts (White Post DC).

For the control group, the posts were immersed in 70% alcohol for one minute to clean the surface and then dried using sterile gauze. A layer of silane (Dentsply Maillefer, Petrópolis, RJ, Brazil) was applied to the surface of the post for one minute.

For the customized group, the post surfaces were cleaned and silanized in the same way as in the control group. The customization was carried out with the direct use of translucent resin composite (CT, Filtek Z350 XT, 3M ESPE, St. Paul, MN, USA). The resin was placed on the surface of the post and the post/resin assembly was inserted into the root canal previously isolated with a water-soluble gel (KY Gel, Johnson & Johnson, São José dos Campos, SP, Brazil), followed by light-activation for 5 seconds, and was then removed and light-activated for a further 40 seconds. The posts were cleaned and silanized once more. The insertion and removal axis was demarcated with a marker pen on the tooth and on the post.

### 2.3. Cementation

Prior to cementation, the root canals were washed in 2 mL of distilled water to remove the water-soluble gel, the final irrigation being done with 1 mL EDTA (Fórmula e Ação) for three minutes. The canals were vacuum-dried using cannulas (Ultradent Products Inc.), the drying being complemented with absorbent paper cones (Dentsply Maillefer).

The self-etching resin cement U200 (3M ESPE Sumaré, São Paulo, Brazil) was handled according to manufacturer's directions and introduced into the root canal with a Centrix syringe (Nova DFL; Rio de Janeiro, RJ, Brazil). The insertion of the post in the root canal was standardized with a parallelometer (Bio-Art Equipamentos Odontológicos Ltda., São Carlos, São Paulo, Brazil), to ensure it is maintained in a central position, parallel to the long axis of the root, while the light-activation was carried out for 60 seconds in the cervical portion of the root, with a photopolymerizer (3M ESPE, Sumaré, São Paulo, Brazil) and an energy dose of 800 mW/cm^2^.

The specimens were kept humid for 7 days at 37°C. After removal from storage, they were submitted for an analysis of the cement line and push-out bond resistance.

### 2.4. Analysis of the Cement Line

**Figure 1:**
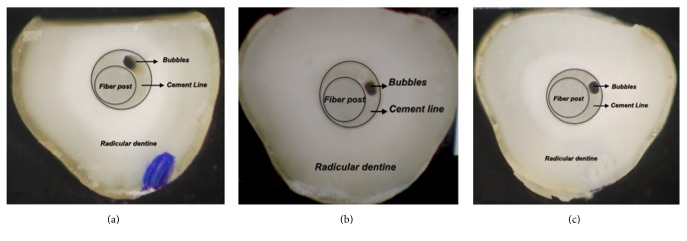
(a), (b), and (c): cervical, middle, and apical slices, respectively, of the control group.

**Figure 2:**
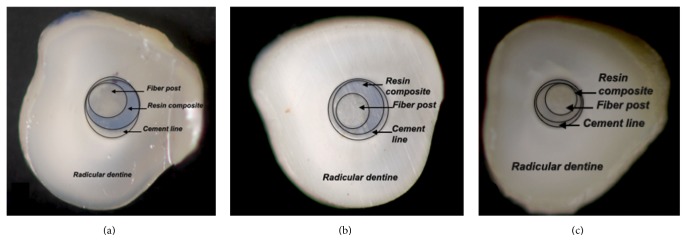
(a), (b), and (c): cervical, middle, and apical slices, respectively, of the customized group.

The roots were sectioned perpendicular to the long axis in three slices measuring 1.2 mm (±0.1 mm) (apical, middle and cervical), with a double-faced diamond disk (Buehler Ltd., Lake Bluff, IL, USA) connected to a universal cutting machine (Isomet Low Speed Saw, Buehler) under constant cooling. Digital images were captured of both sides of the slices using a digital camera (Q-Color5, Olympus) connected to a stereomicroscope loupe (SZ61, Olympus America Inc., PA, USA), using 30x magnification (Figures 1, 2(a), 2(b), and 2(c)).

**Figure 3:**
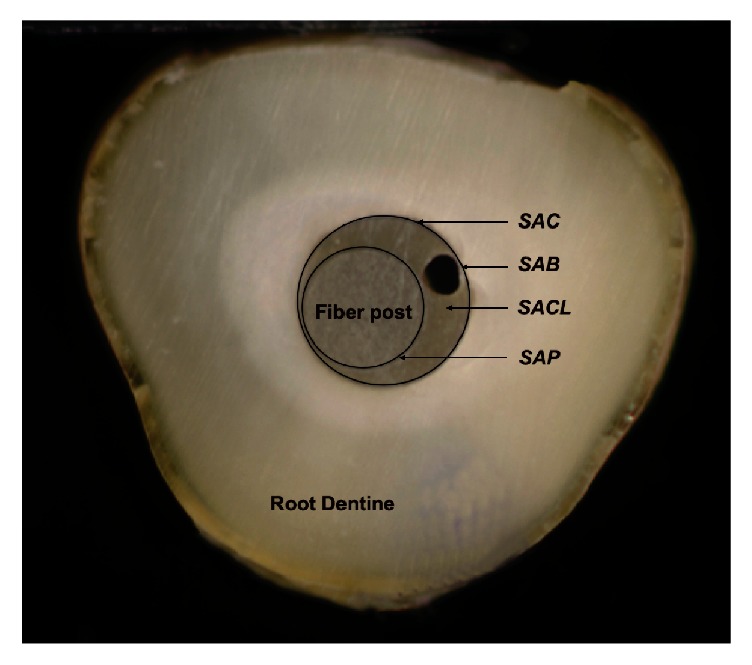
Surface area of root canal (SAC); surface area of post (SAP); subtraction of SAP from SAC, establishing the surface area of the cement layer (SACL); surface area of air bubbles present (SAB).

The photomicrographs obtained were analyzed using the ImageJ software application (National Institute of Health, Maryland, USA - https://rsb.info.nih.gov/ij/). For this, the following areas were demarcated: Surface area of root canal (SAC); Surface area of post (SAP); Subtraction of SAP from SAC, establishing the surface area of the cement layer (SACL); Surface area of air bubbles present (SAB); Subtraction of SAB from SACL, establishing the surface area of the cement surface without air bubbles (Figure 3).

Using these measurements, the uniformity of the cement layer was evaluated through the percentage of the areas with and without bubbles.

### 2.5. Analysis of Bond Strength

The push-out test was conducted using a universal testing machine (EMIC, Instron Brasil Equipamentos Científicos Ltda., São José dos Pinhais, PR, Brazil), using the previously obtained root slices. The thickness of the slices was measured using a digital caliper with a resolution of 0.01 mm (Mitutoyo MTI Corporation, Tokyo, Japan). Each slice was placed on a push-out device (ODEME, Luzerna, Santa Catarina, Brazil) consisting of a steel base with a 3 mm aperture and a stainless steel punch 1 mm in diameter. The punch exerted a downward force at a velocity of 0.5 mm/min, until the post was completely extruded.

The bond strength of each slice was calculated as the force (N) divided by the bonded cross-sectional surface area and expressed in MPa. The bonded area of each section was calculated using the following formula: *π* × (*r*_1_ + *r*_2_)×√(*r*_2_ − *r*_1_)^2^ + *h*^2^, where *π* is the constant 3.14, *r*_1_ and *r*_2_ are the smaller and larger radii, respectively, and *h* is the height of the section in mm.

The pushed-out specimens were cleaved longitudinally and the root segments were observed under 40x magnification with a stereomicroscope loupe The failures were classified as follows: adhesive between the cement and post (ACP), adhesive between cement and dentin (ACD), and mixed (M) and cohesive in dentin (CD).

### 2.6. Statistical Analysis

The data were analyzed using SAS software (Version 9.0; SAS Institute Inc., Cary, NC, USA). The normal data distribution was investigated beforehand, the data being transformed according to the suggestion provided by the software.

For the analysis of bond strength, the two-way ANOVA test was employed followed by the Tukey test.

A univariate linear regression model was put together to estimate the influence of the area occupied by bubbles in the cement layer on the bond strength, measured by means of coefficients of determination (*R*^2^) and regression (*β*). The correlation between these variables was also investigated using Spearman's coefficient. The level of significance employed in all the tests was 5%.

## 3. Results

The data presented statistically significant differences between the groups, in the different root thirds in relation to the area occupied by the air bubbles (*p* < 0.05). Moreover, it was found that the middle third was the region most affected by bubbles, in both groups (Table 1).

Bond strength, taking into consideration all the thirds, was 8.77 ± 4.89 MPa for the control group and 16.96 ± 4.85 MPa for the customized group. There were statistically significant differences between the groups, regardless of the root third evaluated (*p* < 0.001) (Table 2).

When the bond strength was correlated with the area occupied by the bubbles, an inverse correlation was found, for both the control group (*R*_*s*_ = −0.725) and the customized group (*R*_*s*_ = −0.682) (Table 3).

As for the specimens' fracture pattern, there was a prevalence of adhesive fractures between the cement and the dentin 77.8% for the control group and 72.2% for the customized group, respectively, followed by cohesive post fracture (22.25% for both groups) (Table 4).

## 4. Discussion

The null hypothesis tested was rejected as there was a significant difference in bond strength between the groups in the study. The customization of the glass fiber post using composite resin increased the bond strength values at the post/resin cement interface in the cervical, middle, and apical root thirds. An increase in bond strength values was observed in the customized group, compared to the control group. This may be attributable to the fact that customization reduces the cement layer and thus allows for better post adaptation, creating a more uniform unit between post, resin, and cement.

The push-out test evaluated the bond strength of the post/cement interface. The literature demonstrates that these bond strength values vary a lot depending on the methodology employed, the adhesion system, and the cements used. [19, 20]. In this study, bond strength was tested with the punch position coinciding with the post. In the customized group, the post/composite resin assembly was considered for the positioning of the punch.

In the methodology used, it was decided not to carry out the filling using gutta-percha, or any endodontic cement, in order to avoid potential impact on adhesion from endodontic treatment residue [21].

By analyzing the cement layer for the two groups evaluated, it was found that the customization of the glass fiber posts was capable of significantly reducing the formation of bubbles in the cement layer (*p* < 0.05), particularly in the apical third (*p* < 0.001), ensuring a more uniform layer than that obtained in the control group.

Analyzing the results of the control group, the highest bond strength values were found in the middle and cervical thirds, a similar behavior to that demonstrated in other studies [22, 23]. This fact can be explained, as the root dentin presents a reduction in the density of the dentin tubules in the cervical to apical third, the apical third being the least favorable to hybridization, with areas without dentin tubules and irregular dentin [24]. Another factor that may be related to lower bond strength values could be ineffective polymerization of the resin cement in the most apical portion, with a lower degree of conversion of resin monomers, preventing the formation of a homogeneous hybrid layer [24].

The customized group showed a different behavior, as the highest bond strength values were found in the apical third. This may be attributable to a reduced resin cement thickness, and therefore there would be a smaller amount of cement, thus a lower polymerization contraction stress, promoting greater mechanical strength in the cement. Moreover, a smaller quantity of air bubbles was found in the apical third in the group of customized posts, which might also explain the higher bond strength in this area.

In all the thirds evaluated in the control group, a greater surface area was occupied by air bubbles, which might also explain the lower bond strength values when compared to the customized group.

The most prevalent failure mode in this study is in agreement with earlier studies and reinforces the assertion that the most sensitive interface is between the dentin and resin cement, as it has the greatest concentration of stress [25].

## 5. Conclusion

Anatomically customized posts demonstrated greater bond strength than noncustomized posts (control group) and permitted a more uniform cement layer.

## Figures and Tables

**Table 1:** Mean and standard deviation of the area occupied by air bubbles (mm^2^) in the different root thirds, between the control group and customized group.

Group		
Thirds	Control	Customized
Cervical	12.38 ± 5.69 (A)	1.25 ± 0.13 (B)
Middle	24.48 ± 8.11 (A)	2.01 ± 0.28 (B)
Apical	13.02 ± 0.68 (A)	0.49 ± 0.09 (B)

Uppercase letters indicate statistically significant difference between groups (one-way ANOVA, Tukey, *p *< 0.05).

**Table 2:** Mean and standard deviation of bond strength (MPa) in the different root thirds, between the groups.

Group		
Thirds	Control	Customized
Cervical	7.78 ± 4.33 (A. a)	13.59 ± 2.07 (B. a)
Middle	11.35 ± 5.78 (A. b)	17.97 ± 6.17 (B, ab)
Apical	7.19 ± 3.08 (A. a)	19.32 ± 3.20 (B, b)

Total	8.77 ± 4.89^*∗*^	16.96 ± 4.85

Uppercase letters indicate statistically significant difference between types of post. Lowercase letters indicate statistically significant difference between the root thirds (two-way ANOVA, Tukey, *p* < 0.05). The (*∗*) symbol indicates a statistically significant difference between the types of post (*p* < 0.05).

**Table 3:** Analysis of Spearman's correlation and regression between uniformity of cement layer and bond strength.

Group	*R*_*s*_	*R* ^2^	*β*	Value *p*
Control	−0.725	0.005	−3.28	<0.001^*∗*^
Customized	−0.682	0.452	+6.95	<0.001^*∗*^

*R*_*s*_: Spearman's correlation coefficient. *R*^2^: coefficient of determination. *β*: coefficient of regression. (^*∗*^*p*< 0.05).

**Table 4:** Frequency and percentage of fracture pattern in each group.

Thirds	Control					Customized				
	1	2	3	4	5	1	2	3	4	5
Cervical	0	5	0	1	0	0	5	0	1	0
Middle	0	4	0	2	0	1	4	0	1	0
Apical	0	5	0	1	0	0	4	0	2	0

Total	0	14	0	4	0	1	13	0	4	0
	0%	77.8%	0%	22.2%	0%	5.6%	72.2%	0%	22.2%	0%

Failure modes: 1: adhesive between post and resin cement; 2: between resin cement and root dentin; 3: mixed, with resin cement partially covering the post surface; 4: cohesive within the fiber post; and 5: cohesive within the dentin.
